# Establishment of Cutoff Values for Newborn Screening of Six Lysosomal Storage Disorders by Tandem Mass Spectrometry

**DOI:** 10.3389/fped.2022.814461

**Published:** 2022-03-28

**Authors:** Ruotong Li, Liping Tian, Qing Gao, Yuanfang Guo, Gaijie Li, Yulin Li, Meng Sun, Yan Yan, Qing Li, Wenying Nie, Hui Zou

**Affiliations:** Neonatal Disease Screening Center, Jinan Maternity and Child Care Hospital Affiliated With Shandong First Medical University, Jinan, China

**Keywords:** lysosomal storage disorders, newborn screening, cut-off value, tandem (hybrid) mass spectrometry, diagnose

## Abstract

**Objective:**

Lysosomal storage disorders (LSDs) are becoming increasingly important in newborn screening, and tandem mass spectrometry (MS/MS) is widely used in newborn screening for LSDs through measurement of enzymatic activities in dried blood spots (DBSs). Overall, the determination of the cutoff value is important in such screening, and different laboratories have different methods of determining this value; most do not use a fixed cutoff value but rather calculate the corresponding batch cutoff value based on each batch of experimental data. In this study, we used MS/MS to screen for LSDs and sought to find an appropriate method to establish the cutoff value for LSD screening.

**Methods:**

A total of 38,945 samples from newborn blood tablets collected from various maternity hospitals in six cities in Shandong province, including Jinan, Dezhou, Heze, Linyi, Weifang, and Zibo, were tested using a *Waters Xevo TQD* tandem mass spectrometer; the experimental data were analyzed with *MassLynx V4.1*. The laboratory used 30% of the median GLA enzyme activity and 20% of the median ABG, ASM, GALC, IDUA, and GAA enzyme activities in every test as the cutoff values for that batch of experiments.

**Results:**

There were 254 suspicious positives in the initial screening test, including one case of Gaucher disease, one of Niemann-Pick disease, 47 of Krabbe disease, four of MPS-I, 21 of Fabry disease, and 180 of Pompe disease. After genetic screening, 11 children were diagnosed, including three with Pompe disease, three with Fabry disease, and five with Krabbe disease. In addition, the enzyme activity cutoff value of this experiment showed seasonal variation, which was initially believed to be related to the ambient temperature, such as the effect of ambient temperature on the human body or the temperature when the blood tablets dried naturally.

**Conclusion:**

Overall, MS/MS can be used in LSD screening, and using different cutoff values in each batch of experiments is feasible. The ambient temperature might be a reason why the enzyme activity cutoff value has seasonal variation. More samples are needed to develop a method of determining cutoff values in laboratories.

## Introduction

Lysosomal storage disorders (LSDs) are a group of rare genetic metabolic diseases, and it is found that early treatment can lead to the optimal clinical effect (1). Some research finds that there is approximately one LSD case among ~7,700 newborns (2). Therefore, newborn screening for LSDs is increasingly important.

Lysosomes are organelles with monolayer membrane cystic structures containing a variety of hydrolytic enzyme substances that decompose many substances. Approximately 60 enzymes are involved in lysosomal catabolism (1). LSDs occur when some of these enzymes or other lysosomal proteins cannot perform their functions. Moreover, LSDs are believed to occur due to genetic variation (3). Indeed, mutation can lead to deficiency in certain lysosomal enzymes, which causes the corresponding substrates in the cells to be stored without being degraded, causing cell metabolism disorders and organ dysfunction (4). Nevertheless, some nonenzymatic lysosomal and nonlysosomal proteins related to lysosomal activity are considered to only represent a small component, and such accumulation might begin early in embryonic development (5). Currently, LSDs comprise more than 50 genetic metabolic disorders. Most of these disorders are inherited in an autosomal recessive manner with a small portion being X-chromosome linked (1). Lysosomal diseases can be classified in many ways, the most common of which is based on the nature of the accumulated substrate. For example, the corresponding enzymes in MPS-I, Fabry, Gaucher, Krabbe, Niemann-Pick, and Pompe diseases are IDUA, GLA, ABG, GALC, ASM, and GAA, respectively. However, some research in Chinese populations shows that the most common LSD in China is MPS-II (6).

The first disease screened for in neonates using dried blood tablets was phenylketonuria (PKU); the detection method reveals whether a neonate lacks phenylalanine hydroxylase by assessing the amount of the enzyme present (7). Previous studies find that the enzymatic activities of several lysosomal enzymes are retained in dried blood spots (DBSs) used for neonatal disease screening (8). Therefore, DBS testing can be used in the screening of LSDs. Tandem mass spectrometry (MS/MS) is currently the most common screening method for DBSs, and it can quantitatively assess lysosomal enzyme activity.

The gold standard for any test is the positive predictive value (PPV), which is the ratio of true positives to test positives (10). However, the determination of this value is difficult for LSD screening experiments because it is difficult to determine whether newborns who screen as positive truly have the disorder. First, some gene mutations lead to late-onset LSDs, such as late-onset Pompe disease, which would delay the determination of true positives (9). Second, LSDs are considered rare diseases, and the incidence of some is one in tens of or even hundreds of thousands (the incidence of MPS-VII could be <1 in 300,000) (11). Thus, a laboratory needs sufficiently large experimental data to prove the PPV. Based on the above, the false negative and positive rates are significant for NBS screening laboratories; determination of the cutoff value is also important. According to some research, most laboratories do not use a fixed enzyme activity cutoff value, but generally use the multiple or percentage of the median or mean of the enzyme activity of a test batch with unique cutoff values for each batch (7). According to the research of [Burlina et al. (12)], using 30% of the median GLA enzyme activity and 20% of the median of the other enzyme activities in each test as cutoff values would result in recalls that neither miss positive cases nor recall too many healthy children. For the first time, we used this method to determine cutoff values, such that the cutoff value could be flexibly adjusted according to the test situation to prevent excessive false positive or negative detection. Furthermore, a stable cutoff value would render data analysis relatively straightforward and allow screening results to be more comparable between studies. In this study, we sought to determine a fixed cutoff value.

This study summarizes 38,945 LSD screening data points, including MPS-I, Fabry, Gaucher, Krabbe, Niemann-Pick, and Pompe disease data obtained by MS/MS, from the Newborn Disease Screening Laboratory in Jinan City. We also verified the test method and the method used to establish the cutoff value.

## Methods

The main technique used for this project was MS/MS, which can determine the activity of six lysosomal enzymes in newborns, allowing for the risk of six LSDs to be assessed.

### Instrumentation

A tandem mass spectrometer (*Waters Xevo TOD)* and incubation shaker (*Thermo MB100-4A*) were utilized in this study.

### Reagents

A screening kit for six LSDs (NeoLSD MS/MS Kit, PerkinElmer) was used in this study.

### Procedure

The samples used in this experiment were DBSs of newborns (age between 27 h and 7 days), which were sent to the Jinan Newborn Disease Screening Center from various maternity hospitals in six cities in Shandong province, including Jinan, Dezhou, Heze, Linyi, Weifang, and Zibo; informed consent was obtained from the parents. The collected dried blood tablets were stored under low-temperature drying conditions. Every week, the experimenter randomly selected ~700 continuous samples from the screening samples for that week, which was convenient for associating newborn information with LSD data for LSD detection. Moreover, we conducted a reexamination of the original blood film of the positive samples in the previous batch of experiments as well as a call-up test for suspected positive cases.

The dried blood tablets were hole-punched into 96-well plates with one hole-punched sample per dried blood tablet. Each 96-well plate included two blank wells (BL), two sets of DBS quality controls (C1, C2, C3), and 88 samples. After adding 30 μl of reaction mixture containing buffer, six substrates, and six internal standards, the plates were covered with an aluminum foil microplate sealing membrane and incubated on a shaker (400 rpm) for more than 18 h at 37°C. After incubation, the microplates were cooled to room temperature, and 100 μl of quencher (a mixture of methanol and NeoLSD extract with a volume ratio of 50:50) was added to stop the reaction; the samples were repeatedly pipetted up and down 10 times with pipette tips to ensure that the blood spot solution was completely mixed with the quencher. Then, all the liquid in the microplates was transferred to deep 96-well plates, and 400 μl of NeoLSD extract (ethyl acetate) and 200 μl of ultrapure water were added to each well with repeated blowing 20 times using a pipette tip. The aluminum foil microplates were sealed and centrifuged for 5 min at 2,500 rpm, after which 50 μl of the supernatant was pipetted into a U-shaped microplate. This step was conducted carefully to avoid collecting any of the lower water layers. The liquid in the microplate was blown dry using nitrogen. After the reagent was completely volatilized, 100 μl of flow solvent was added, the sealing film was attached, and the mixture was shaken at low speed for 10 min prior to analysis.

The analysts checked whether the quality control and total ion chromatogram (TIC) were normal and uploaded them to the Anaconda system to analyze the resulting data. For cutoff value establishment, the laboratory initially established the value based on the research of Burlina et al. (12), in which the cutoff value of GLA enzyme activity was 30% of the median activity in each test, and 20% of the median of IDUA, ABG, ASM, GALC, and GAA enzyme activities in each test was used as the cutoff values for these enzymes. If the first screening result was positive, the analysts performed a reexamination of the original DBS. Then, if the results of both tests were positive, the center recalled the newborns for a second blood sampling and DBS test. Finally, if the results of reexamination were positive, the child underwent DNA tests based on the doctors' judgment.

## Results

### Summary of Screening Experimental Results

There were a total of 38,945 newborns who were screened for six LSDs by MS/MS using DBSs. If the results of the first newborn screening were positive, the original DBS was used for a second test. The newborns with two positives in screening were recalled to collect a new DBS for analysis. If the results of the review were positive, doctors combined the children's physical signs and genetic test results as confirmation of the diagnosis. Table 1 shows the summary of the positives in the initial screening, positives in the original DBS reviews, positives in the second DBS reviews and confirmed number of cases. For all DBSs in LSD screening, there were 402 positives in the initial test, and 254 samples were still positive based on the original DBS review. Among them, GAA had the highest positive rate. For the children who were still positive in the original DBS reviews, the NBS center recalled them and collected a second DBS for testing. Finally, the children were subjected to genetic screening, and three children were diagnosed with Pompe disease, three were diagnosed with Fabry disease, and five were diagnosed with Krabbe disease (Table 2). Additionally, the genetic results revealed five Pompe disease carriers, two Fabry disease carriers, and three Krabbe disease carriers.

**Table 1:** The rate of screening positive, review positive, and confirmed disorders in LSD newborn screening (due to lack of reagents, not all the initial screening positives on July 22 and July 27, 2020, were followed up).

**Enzyme/LSD**	**ABG**	**ASM**	**GALC**	**IDUA**	**GLA**	**GAA**	**Total**
Positives in initial screening	12	1	71	5	34	279	402
Positives in second original DBS review	1	1	47	4	21	180	254
Suspected cases	0	0	5	0	3	3	11

*The type of gene sequence variation in nine suspected cases was “uncertain significance” but with a persistent decrease in enzyme activity as well as signs*.

**Table 2:** Genetic site information and enzyme activity in the screening experiment in confirmed cases.

**Number**	**Disease**	**Enzyme**	**Genetic results**	**Screening result**
1	Fabry	GLA	c.1019G > C	0.3 (2.54)
2	Fabry	GLA	c.1286–7del	2.12 (2.74)
3	Fabry	GLA	c.454T > C	0.48 (2.56)
4	Pompe	GAA	c.859-2A > T c.2065G > A	0.42 (1,73)
5	Pompe	GAA	c.752C > T c.761C > T	0.61 (1.92)
6	Pompe	GAA	c.752–761delins c.1757C > T	0.4 (1.72)
7	Krabbe	GALC	c.1901T > C c.2041G > A	0.33 (0.93)
8	Krabbe	GALC	c.1901T > C c.1901T > C	0.52 (0.92)
9	Krabbe	GALC	c.1901T > C c.1901T > C	0.45 (0.87)
10	Krabbe	GALC	c.1204G > A c.1535G > T	0.21 (0.7)
11	Krabbe	GALC	c.1901T > C c.1671–14G > A	0.35 (0.61)

*In the “screening result” column, the parentheses are cutoff values for the batch of experiments*.

### Cutoff Value

**Figure 1:**
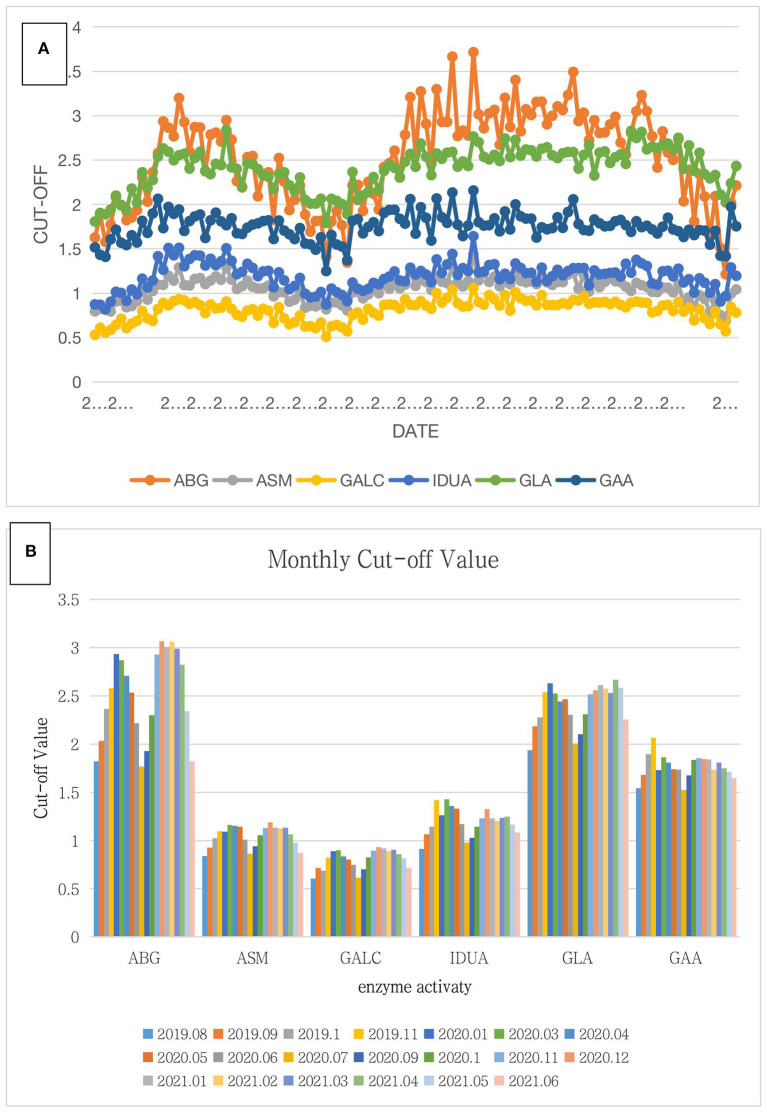
Summary of the cutoff value for 38,945 screening samples. **(A)** In this chart, the abscissa is the date of the experiment, and the ordinate is the cutoff value of six lysosomal enzymes. The orange, gray, yellow, blue, green, and navy blue lines represent ABG, ASM, GALC, IDUA, GLA, and GAA, respectively. The overall trend of the six lysosomal enzymes was first an increase and then a decrease followed by another increase, finally showing a downward trend. Among them, the increasing trends of ABG and GLA were obvious though the changes in ASM, IDUA, GAA, and GALC were not significant. **(B)** In this chart, the abscissa is six lysosomal enzymes, and the ordinate is the average monthly cutoff value. Differently colored bars represent different months. For all six lysosomal enzymes, the average cutoff values were the lowest in July and highest in November, December, January, or February.

ABG, ASM, GALC, IDUA, and GAA are lysosomal enzymes with reduced activity in Gaucher disease, Nimann-Pick disease, Krabbe disease, MPS-I, and Pompe disease, respectively. Thus, we decided to use 30% of the median enzyme activity of GLA in each experiment as the cutoff value for Fabry disease; the cutoff values for the other five diseases were 20% of the median enzyme activity. To determine the fixed cutoff value, the cutoff values for all experiments from August 2019 to June 2021 were summarized (Figure 1). Activities of ABG and GLA lysosomal enzymes changed significantly over time, peaked in November 2019 and December 2020, and decreased to a minimum in July 2020. In contrast, the other four lysosomal enzymes did not show a significant seasonal change (Figure 1A). Figure 1B compares the monthly average cutoff values after statistical analysis of the data during the experiment. All six lysosomal enzymes showed the lowest activity in July, whereas the highest activity appeared in November, December, January, or February.

## Discussion

It is necessary to promote newborn screening for LSDs. In Europe, the United States, Taiwan, and many other countries, this screening has been ongoing for a long time (12). MS/MS, a high-throughput screening method, can detect enzyme activity by measuring the number of related products of the reaction (13). Moreover, the application of MS/MS to disease screening is common in neonatal disease-screening laboratories; it was first used to screen for PKU. In this study, MS/MS was mainly used to analyze the activity of IDUA, ABG, ASM, GALC, GLA, and GAA, which are reduced in MPS-I, Gaucher disease, Nimann-Pick disease, Krabbe disease, Fabry disease, and Pompe disease, respectively. Among these 38,945 samples, three children were diagnosed with Pompe disease, three with Fabry disease, and five with Krabbe disease through genetic screening. The total incidence of LSDs was nearly 1/3,000 though some research shows that the rate of these diseases is 1/6,000–7,000 (2). This discrepancy might be due to the inclusion of carriers in screen-positive specimens, and the enzyme activity of carriers would also be reduced. More samples are needed to verify the disease incidence in Shandong. The positive rate in Pompe disease was highest among the six diseases in the screening experiment, but the number of diagnosed children was not the largest either due to the effect on screening results of reduced enzyme activity in carriers or because current cutoff value establishment methods are not suitable for Pompe disease; more samples are needed to clarify this. On the other hand, there were no confirmed cases of Gaucher disease, Niemann-Pick disease, or MPS-I among any of the test samples. The incidence of Gaucher disease has strong regional and ethnic differences; it is relatively common in Eastern Europe (14), and the incidence is ~1/500,000 to 1,000,000 (15). Similarly, Niemann-Pick disease has the lowest incidence in China, and it is mainly found in the Middle East, Western Europe, North America, and other countries, which may be why no confirmed cases of these two diseases were found in this study. Although some research shows that MPS is common in China, there were no confirmed cases in this study. This result might be because MPS with a relatively high incidence in China would be type II or type IVA (16), but the main MPS type analyzed in this study was MPS-I. Therefore, research on MPS-II and MPS-IV marker screening tests is needed.

We sought to determine a fixed cutoff value of the LSD screening experiment for this laboratory. However, lysosomal enzyme activity cutoff values differed according to the month. In fact, lysosomal enzyme activity cutoff values were lowest in July and August and highest in December and January. This situation made it difficult to establish a fixed cutoff value. We attempted to explore the reasons for this, and we initially thought changes in temperature to be a factor. The main principle of this experiment was to detect the activity of lysosomal enzymes in DBSs, and enzyme activity appears to be affected by temperature (17). This effect might be divided into the effect of low or high temperatures, but the main difference between these two conditions is that high temperatures would completely inactivate enzymes and low temperatures would not (18). This research was conducted in Shandong, China, and July and August are the 2 months with the highest average temperature in this region. Moreover, all six lysosomal enzymes showed low activity, which might be affected by high temperature. Conversely, low temperature would not inactivate an enzyme but cause a dormant state with activity being restored when the temperature returns to a suitable one (19). The results of this experiment are in accordance with this idea. All six lysosomal enzyme activities were highest in December and January, when the temperature was lowest in the 1-year test. This result might be because a relatively low ambient temperature would provide low-temperature storage conditions for lysosomal enzymes, which would better retain the enzyme activity. This is in accordance with the change in enzyme activity. Therefore, the change in the cutoff value might be related to the change in temperature. This experiment was carried out in a laboratory environment with constant temperature and humidity, and the blood tablets were transported to the laboratory through a cold chain. Nonetheless, the effect of different outdoor temperatures on enzyme activity in the human body and blood tablets at room temperature drying after collection might also cause changes in enzyme activity. In summary, using a fixed cutoff value leads to an increase in false positives in winter and false negatives in summer. However, more samples are required to further verify the effectiveness of the cutoff value establishment method. Although the incidence of lysosomal storage disease is 1 in 7,000 to 8,000, the incidence of each single disease is relatively low; for example, the incidence of Pompe disease is ~1 in 40,000 (20).

Overall, MS/MS is feasible for screening LSDs. In this research, the highest initial screening positive rate was for Pompe disease (GAA), and there were eight children with suspected related gene mutation among the 38,945 newborns in the newborn screening test of LSDs but only three confirmed cases with two causative sites were detected. Moreover, we found that the lysosomal enzyme activity cutoff value exhibited seasonal variation, and we suggest that the main reason for this is the effect of seasonal temperature on enzyme activity. Therefore, it is optimal to change the cutoff value for each test batch. According to the current experimental data, the cutoff value establishment method was feasible, but the high rate of positivity in Pompe disease screening might reflect that this cutoff value establishment method is not suitable for screening for Pompe disease in this laboratory. More samples are needed. Overall, 11 children with suspected mutations were successfully screened in this study. However, due to the low disease incidence, more samples are needed for further verification. Furthermore, as LSDs with high incidence in China are different from those in other countries, new markers need to be further studied to improve screening of LSDs in the Chinese population.

## Data Availability Statement

The original contributions presented in the study are included in the article/supplementary materials, further inquiries can be directed to the corresponding authors.

## Author Contributions

RL and LT designed the study and wrote the manuscript. RL, QG, YG, GL, YY, and QL prepared the materials used in the experiment and collated the data. RL, YL, and MS analyzed the data. HZ and WN were responsible for the supervision and leadership of the experiment and the review of the first draft of the manuscript. All authors contributed to the article and approved the submitted version.

## Funding

This work was supported by the Shandong Provincial Natural Science Foundation (2021.01-2023.12).

## Conflict of Interest

The authors declare that the research was conducted in the absence of any commercial or financial relationships that could be construed as a potential conflict of interest.

## Publisher's Note

All claims expressed in this article are solely those of the authors and do not necessarily represent those of their affiliated organizations, or those of the publisher, the editors and the reviewers. Any product that may be evaluated in this article, or claim that may be made by its manufacturer, is not guaranteed or endorsed by the publisher.
